# Inelastic scattering and solvent scattering reduce dynamical diffraction in biological crystals

**DOI:** 10.1107/S2052520619009661

**Published:** 2019-08-01

**Authors:** Tatiana Latychevskaia, Jan Pieter Abrahams

**Affiliations:** a Laboratory of Nanoscale Biology, Paul Scherrer Institute, Forschungsstrasse 111, Villigen, 5232, Switzerland; b Biozentrum, Basel University, C-CINA, Mattenstrasse 26, Basel, 4058, Switzerland; c IBL, Leiden University, Sylviusweg 72, Leiden, 2333 BE, The Netherlands

**Keywords:** electron diffraction, multislice calculation, inelastic electron scattering, protein crystallography, electron crystallography, cryo-EM

## Abstract

Multi-slice simulations of electron diffraction by three-dimensional protein crystals have indicated that structure solution would be severely impeded by dynamical diffraction, especially when crystals are more than a few unit cells thick. In practice, however, dynamical diffraction turned out to be less of a problem than anticipated on the basis of these simulations. Here it is shown that two scattering phenomena, which are usually omitted from multi-slice simulations, reduce the dynamical effect: solvent scattering reduces the phase differences within the exit beam and inelastic scattering followed by elastic scattering results in diffusion of dynamical scattering out of Bragg peaks. Thus, these independent phenomena provide potential reasons for the apparent discrepancy between theory and practice in protein electron crystallography.

## Abstract 2

Multiple, dynamical electron diffraction can be simulated by multislice calculations. However, experimental evidence indicates that these calculations overestimate dynamical diffraction. It is shown that ignoring bulk solvent scattering and inelastic scattering in calculations, results in overestimating dynamical effects.

## 1. Introduction   

The 2017 Nobel Prize in Chemistry for Henderson, Frank and Dubochet who were key contributors to the development of cryo-electron microscopy (cryo-EM) for acquiring three-dimensional atomic, structural information of biological complexes, confirmed the enormous impact of methods that employ electron scattering for structure determination.^1^ In cryo-EM, the scattered electrons are imaged on a direct electron detector by a series of electron lenses, yielding a projection image of the scattering molecular complex. Because of the limited total electron dose that a biological sample can tolerate, the phase contrast of such a sample is weak and image quality is degraded by electron-optical distortions and potential drifts during the exposure, the signal-to-noise ratio (SNR) of cryo-EM data is poor at high resolution.

### 1.1. Mismatch between the theory and experiment   

**Figure 1:**
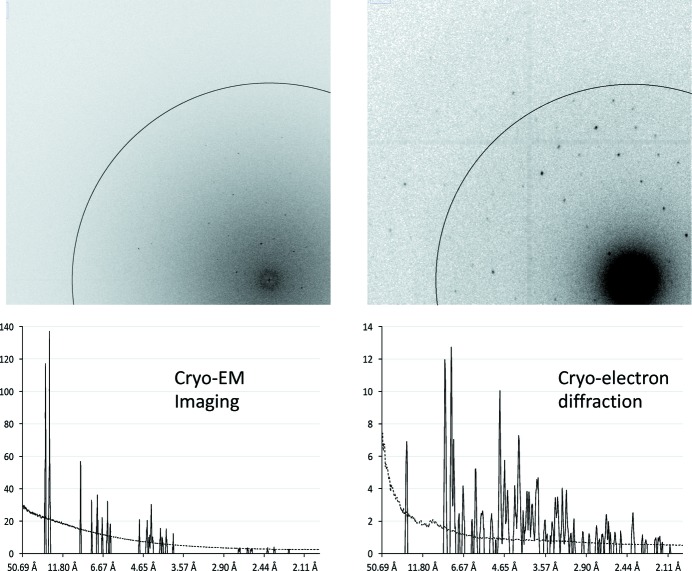
Imaging lysozyme nanocrystal in imaging and diffraction mode. The top-left panel shows the Fourier transform of a 300 keV electron image of a ±100 nm-thick lysozyme nano-crystal (measured at Scherzer focus) collected using a Titan Krios EM with a Falcon direct electron detector (Nederlof *et al.*, 2013 ▸). The illuminated crystal volume was about 400 nm × 200 nm × 100 nm and the electron dose was about 6 e^−^ Å^−2^. The lower-left panel indicates the signal-to-noise ratio of this imaging data. The circle indicates 3 Å resolution. A peak search routine identified potential Bragg spots above background (van Genderen *et al.*, 2016 ▸). The average peak height of these spots was plotted as a function of resolution with a solid line. The average diffuse background was plotted with a dotted line. The top right panel shows a diffraction pattern of a lysozyme nanocrystal. The crystal had a very similar size to the crystal of the left panel and had the same space group. The 200 keV data were collected using a Titan Krios EM equipped with a Medipix direct electron detector. The diffracted crystal volume was about 1000 nm × 200 nm × 100 nm and the electron dose was 0.06 e^−^ Å^−2^. The lower-right panel indicates the plot which was calculated in a similar way to the corresponding lower-left plot of the imaging data.

Measuring electron scattering data from protein crystals in diffraction mode, rather than in imaging microscopy mode, circumvents or reduces several of the phenomena that compromise the signal-to-noise ratio in cryo-EM. Thus, measuring in diffraction mode can result in a reduction by orders of magnitude of the electron dose required for achieving high resolution when imaging three-dimensional protein crystals. Analysis of published data for lysozyme nanocrystals of similar size and identical space group in either imaging or in diffraction mode under optimal conditions (Nederlof *et al.*, 2013 ▸; Clabbers *et al.*, 2017 ▸) indicates that diffraction data requires an electron dose that is about three orders of magnitude lower to yield a similar Bragg signal by imaging (Fig. 1 ▸). The top-left panel in Fig. 1 shows the Fourier transform of a lysozyme nano-crystal (Nederlof *et al.*, 2013 ▸). Subsequent images of the same crystal still diffracted, but high-resolution spots were already noticeably fading (data not shown). The resolution of the Bragg spots was isotropic, indicating that there was no noticeable beam - or sample drift. The plot in the lower-left panel in Fig. 1 indicates the signal-to-noise ratio of this imaging data. Significant data up to about 3.7 Å resolution could be distinguished unequivocally. The small peaks at higher resolution are most likely due to noise fluctuations. The top right panel in Fig. 1 ▸ shows a diffraction pattern of a lysozyme nano-crystal from a three-dimensional electron diffraction data set comprising about 30° of rotation data (Clabbers *et al.*, 2017 ▸). The plot in the lower-right panel in Fig. 1 ▸ was calculated similarly to the plot of the imaging data. Significant diffraction data to 2.1 Å were clearly visible, despite a total electron dose that was 100 times lower than in the imaging data.

Recently, this difference in data quality was explained from first principles, indicating that the combination of several independent fundamental effects, rather than technological limitations, underlie this observation (Clabbers & Abrahams, 2018 ▸). These fundamental effects include loss of coherency within the sample due to inelastic events, Ewald sphere curvature, signal loss in imaging of samples with a thickness of 50 nm and higher due to focal spread, independency of Friedel mates in diffraction (but not in imaging) and chromatic dispersion due to momentum loss upon elastic scattering. For protein crystals, but likely also for non-crystalline biological samples, there are therefore clear benefits of collecting data in diffraction mode. Yet the point has been made in the literature by several key experts in the field that such data would be severely compromised by multiple or dynamical diffraction (Glaeser & Downing, 1993 ▸; Subramanian *et al.*, 2015 ▸). Multislice calculations indicated that dynamical diffraction would invalidate retrieving the phase information – that is lost in diffraction data, but that is essential for structure interpretation – by conventional crystallographic methods, which are based on kinematic diffraction theory and therefore assumes single elastic scattering. A prime hallmark of dynamical diffraction of a weak phase object is that Friedel pairs can have very different intensities, unlike in kinematic, single scattering diffraction data. Multislice calculations indicated such differences to occur even at modest crystal thickness (Glaeser & Downing, 1993 ▸). Nevertheless, protein structures have been solved by conventional phasing methods using electron diffraction data of relatively thick crystals (*e.g.* Hattne *et al.*, 2015 ▸; Clabbers *et al.*, 2017 ▸), that had differences between Friedel pairs smaller than anticipated on the basis of earlier predictions based on multislice calculations (Glaeser & Downing, 1993 ▸). We therefore concluded that the theoretical analysis must have been incomplete.

### 1.2. Scattering events described by particle and wave models   

High-energy electrons (usually 80 keV and up) are scattered elastically through Coulomb attraction by the nucleus and inelastically scattered through Coulomb repulsion by the inner and outer-shell electrons (Egerton, 2011 ▸). According to a classical (particle) description, upon elastic scattering, the electron can change its direction, but loses virtually no energy, whereas upon inelastic scattering it hardly changes direction, but loses energy and coherency. The probability of scattering is determined by the atomic scattering cross section. The mean free path for electron (elastical or inelastical) scattering describes the average distance which an electron travels without being scattered and it is given by Λ = *A*(*N*_A_ρσ)^−1^, where *A* is the atomic weight, ρ is the density, *N*_A_ is the Avogadro number and σ is the total (elastic or inelastic, respectively) scattering cross section. Alternatively, treating electron as a wave, the scattering process can be described by the complex-valued scattering amplitudes. The differential cross-section dσ/dΩ = |*f*(θ, φ)|^2^ gives the probability that an electron is scattered into the solid angle dΩ, where *f*(θ, φ) denotes the scattering amplitude for the direction determined by the polar angle θ and azimuth φ and which is found by solving the Schrödinger equation. The scattering amplitudes *f*(θ, φ) for elastic scattering have typically a much broader distribution that that for inelastic scattering. The total scattering cross-section σ is obtained from the differential cross section by integrating over the solid angle. The intensity distribution of a scattered wave in the far-field gives the distribution of probabilities of detecting an electron at a selected location on the detector.

### 1.3. Importance of accounting for inelastic scattering   

Henderson (1995 ▸) indicated the importance of considering inelastic scattering in electron microscopy of biological specimens in terms of radiation damage. Here we extend this analysis to include its effect on the quality of the elastic signal.

Like kinematically scattered electrons, dynamically scattered electrons are diffracted in directions determined by the wavelength of the diffracted radiation, the reciprocal lattice parameters and the crystal orientation. Since these values do not change upon multiple elastic diffraction, both kinematic and dynamic diffraction are focused into Bragg spots. However, the situation may change if at least one of the multiple scattering events was inelastic. Then the electron lost some energy, resulting in a longer wavelength and loss of coherency.

The ratio of inelastic scattering can be approximated as *Z*/20 (Egerton, 2011 ▸, p. 126), where *Z* is the atomic number. Hydrogen is a particularly strong inelastic scatterer of electrons and composes more that 50% of the atoms of a biological sample. In a previous analysis of inelastic scattering, biological sample were treated as all-carbon leading to an inelastic *versus* elastic scattering ratio of 3:1 (Henderson, 1995 ▸), but a calculation based on the true atomic composition of hydrated protein samples, indicated a ratio of 4:1. Please note that ignoring inelastic scattering in multislice calculations is not equivalent to filtering out inelastically scattered electrons using an energy filter. For instance, if we ignore inelastic scattering in our calculations, all electrons emerge from a thick sample being scattered dynamically, resulting in highly dynamical diffraction data. In reality, however, virtually all electrons will have scattered inelastically at least once, and would be removed by the energy filter, resulting in no diffraction data whatsoever.

### 1.4. Importance of accounting for multiple electron scattering   

Both elastic and inelastic scattering cross sections are relatively high for electrons, therefore multiple electron scattering (‘dynamical diffraction’) can occur even when the sample is thin.

Protein crystals contain on average about 50% disordered solvent. Because it is disordered, it will not contribute significantly to higher resolution crystal diffraction, but instead give rise to a diffuse, mostly radially symmetrical diffraction signal. The signal has therefore frequently been ignored for studies at high resolution, since its signal can be considered to be part of the background when integrating Bragg spots. Here, we make the point that the diffraction signal of the solvent scattering potential cannot be ignored in multislice calculations. An underlying assumption of the statement that in kinematic, single scatter electron diffraction, the Friedel pairs must have similar intensities, is that the sample is a weak phase object. The essential feature of a weak phase object is that it only introduces a small additional phase φ into the travelling electron wavefunction. The transmission function of the weak phase object be approximated as exp(*i*φ) ∝ 1 + *i*φ. However, by ignoring the phase shift caused by the disordered solvent, this principle is violated. For instance, when the interprotein cavities align with the electron beam, the assumption that they do not contain solvent leads to a zero phase shift at these locations, and as a result to a strong phase shift difference between the locations occupied by the protein molecule and cavities. However, in reality the cavities are filled with solvent, and therefore induce a phase shift comparable to the phase shift experienced by the beam passing through protein. So, ignoring the solvent potential incorrectly converts a protein crystal from a weak phase object into a strong phase object. This exaggerates the differences within Friedel pairs.

Below we provide simulations which include two important phenomena that are not usually included in calculations: inelastic scattering and bulk solvent diffraction.

## 2. Simulations   

### 2.1. Effects of inelastic scattering   

In this section we treat electron scattering as a particle phenomenon. In elastic scattering, the electron can change direction, whilst its wavelength remains constant within measuring accuracy (assuming the particle interpretation). In inelastic scattering however, the electron hardly changes direction, yet it loses some of its energy [on average about 40 eV for high-energy electrons – see Egerton (2011 ▸) for a full discussion]. For instance, at 300 kV, the scattering curve of an inelastic electron is about two orders of magnitude narrower than that of an elastically scattered electron. Thus, after an electron has scattered elastically, any subsequent inelastic scattering events will hardly change its scattering angle. For crystal diffraction, this means that electrons that only scattered inelastically end up in the direct beam. Electrons that scattered elastically and subsequently scattered one or more times inelastically, still end up in the Bragg peak, as they hardly changed direction. So, for these electrons, inelastic scattering results in a small peak broadening. However, this does not mean that inelastic scattering does not affect diffracted amplitudes.

**Figure 2:**
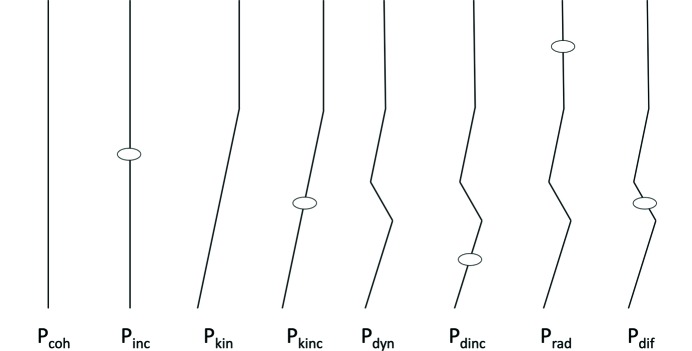
Several types of multiple scattering that each have a different effect on the measured diffracted intensities. Elastic events are indicated by a change in direction, inelastic events are indicated by ovals. Each combination has an associated probability, determined by the elastic and inelastic scattering cross sections and the thickness of the sample*.*

Inelastic scattering is not a coherent event. Concomitant with the energy loss upon inelastic electron scattering, the electron therefore also loses coherency. It can be described by a wavefront with a strongly pronounced amplitude in the direction of the incident electron originating from the location of the inelastic event. The consequence of this loss of coherency, is that subsequent elastic scattering is no longer coherent with elastically scattered electrons. So elastic scattering that occurs after an inelastic event is no longer confined to Bragg spots, as its wavefunction no longer interferes laterally with the sample, but only along the direction of the electron. Therefore, its probability distribution has become radially symmetric about an axis defined by the direction of the last elastic scattering event. For the subsequent discussion we distinguish several combinations of elastic and inelastic scattering events illustrated in Fig. 2 ▸.

Deviations from translational symmetry or phonons cause incoherencies within the sample, resulting in diffusion of the diffraction data out of Bragg spots. Our discussion focuses instead on incoherencies of the beam, that are induced by the sample (which could be fully or only partially ordered). So, the results of our simulation would not necessarily be equivalent with multislice simulations that model diffuse scatter using a ‘frozen phonon’ algorithm (Loane *et al.*, 1991 ▸), as in this algorithm, the electrons remain coherent.

For a disordered sample, the probabilities of these various types of multiple scattering can be calculated by assuming the scattering to be a Poisson process, that is exclusively determined by atomic scattering cross sections. Such a Poisson process requires scattering to have time interval invariance: the probability of an electron being scattered should be independent of whether or when it has already been scattered before. This assumption is clearly not valid when atoms line up along a crystal lattice in tight columns parallel to the electron beam, such is the case for crystals with small unit cells that are aligned with the electron beam. In that case, if an electron encounters an atom in the column, it will certainly be encountering another atom further down, since high-energy electron scattering amplitudes have a narrow angular distribution. This results in a process that has been referred to as electron channelling and enhances the dynamic effects. In imaging, it results in a thickness dependent modulation of observed image intensity at the projected positions of aligned columns (*e.g.* Howie, 1966 ▸; Op de Beeck & Van Dyck, 1996 ▸).

However, in crystals of biological molecules, the atoms do not line up. This allows us to model the probability of scattering *P*_s_(*z*) after traversing a sample over a distance *z* (in nm) as an exponential decay:
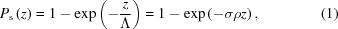
where σ is the average total atomic scattering cross section (in nm^2^) and ρ is the density (in atoms per nm^3^).

The total elastic atomic scattering cross sections σ_e_(*Z*) can be approximated as a function of the atomic number *Z* by an equation based on the Lenz model for electron scattering [see for instance Egerton (2011, p. 115 ▸) for further details on the equations obtained with the Lenz model]:
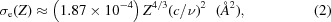
where *c* is speed of light and ν is relativistic speed of the traversing free electron.

The total inelastic scattering cross section σ_i_(*Z*) can conveniently and with reasonable accuracy be approximated from σ_e_(*Z*) according to Egerton (2011 ▸):
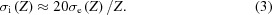



We assumed an average specific density for a protein of 1.35 g ml^−1^ and an average fractional atomic composition of protein to be H_0.48_C_0.32_N_0.09_O_0.09_S_0.02_. The number of molecules in nm^3^ can be evaluated as *N* = ρ /*m*, where *m* is the mass of one molecule, calculated as *m* = *M*/*N*_A_, here *M* is the molar mass and *N*_A_ is the Avogadro number. The calculation shows that a protein on average contains about 106 atoms per nm^3^ (including hydrogens). Since proteins are hydrated, the contribution of solvating water also needs to be considered. Water has a fractional atomic composition of H_0.67_O_0.33_ and a density of about 101 atoms per nm^3^. The fractional atomic composition of a protein crystal containing 50%(*v*/*v*) solvent – which is the average for protein crystals (*e.g.* Kantardjieff & Rupp, 2003 ▸) – therefore is: H_0.57_C_0.17_N_0.05_O_0.20_S_0.01_. Similar considerations lead to a fractional atomic composition for nucleic acid of H_0.52_C_0.15_N_0.06_O_0.20_P_0.02_.

Given this fractional atomic composition, it is straightforward to calculate the total elastic and inelastic elastic scattering cross sections of the average atom of a biological sample by taking the weighted average of its composing atomic scattering total cross sections as inferred from equations 2 and 3 (Table 1 ▸). These values indicate that biological samples tend to scatter less elastically than was presumed in an earlier analysis: only one in five scattering events is elastic, instead of one in three. The main reason for this discrepancy is that biological samples contain significant amounts of hydrogen atoms, for which only one in twenty scattering events is elastic, and that the earlier analysis approximated a biological sample as only containing carbon.

Using the atomic elastic and inelastic scattering factors for the average biological atom, the probabilities of (multiple) elastic and inelastic scattering as a function of sample thickness can now be calculated from a system of differential equations. First, we define:

*P*_e_(d*z*) = σ_e_ρd*z* is the probability of scattering elastically over a distance d*z*.

*P*_i_(d*z*) = σ_i_ρd*z* is the probability of scattering inelastically over a distance d*z*.

*P*_t_(d*z*) = *P*_e_(d*z*) + *P*_i_(d*z*) is the total probability of scattering over a distance d*z*.

Multiple scattering in an amorphous material can be modelled analytically using a Poisson distribution (*e.g.* Childs & Misell, 1972 ▸; Egerton, 2011, p. 175 ▸):
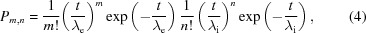
where *P*_*m*,*n*_ is the probability that a transmitted electron suffers *m* elastic *n* inelastic collisions, *t* is the thickness, λ_e_ and λ_i_ are the elastic and inelastic mean free paths, respectively. However, this equation is independent of the order of the elastic and inelastic events, while this order is relevant for the spatial distribution of the observed diffraction. So instead of using an analytic approach, we used the finite difference method that can take the order of the events into account.

The probabilities of the various types of single and multiple scattering as illustrated in Fig. 2 ▸ can now be calculated as follows:
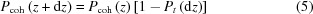
is the probability that the electron is scattered neither elastically nor inelastically at a sample depth of *z* + d*z*. Such unscattered electrons end up in the direct beam.



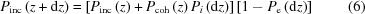
is the probability that electrons that scattered only inelastically (at least once). Previously unscattered electrons that scatter inelastically within the slice d*z* contribute to *P*_inc_(*z*). Electrons that have only scattered inelastically, but that scatter elastically within the slice d*z* reduce *P*_inc_(*z*). Electrons that only scattered inelastically end up in the central beam.



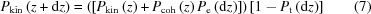
is the probability that electrons that scattered elastically once. Such electrons end up in a Bragg spot and can be used for structure solution using established, kinematic crystallographic theory.

Previously unscattered electrons that scatter elastically within the slice d*z* contribute to *P*_kin_(*z*). Kinematically scattered electrons that scatter a second time within the slice d*z* reduce *P*_kin_(*z*). Depending on the type of the second scattering event, the inelastic or elastic, probabilities *P*_kinc_ or *P*_dyn_ result (see also Fig. 2 ▸):
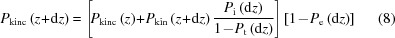
is the probability that kinematically diffracted, incoherent electrons first scattered elastically one single time, and after a subsequent inelastic event are no longer coherent. The probability of inelastic scattering *P*_i_(d*z*) is scaled by [1 − *P*_t_(d*z*)]^−1^ to correct for additional scattering within the slice d*z*. Interaction with the crystal lattice was therefore coherent only up to the depth of the inelastic event. It is therefore as if such electrons experienced a thinner crystal. They will end up in a Bragg spot and can be used be used for structure determination.



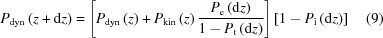
is the probability that dynamically scattered, coherent electrons scattered multiple times, always elastically. They end up in a Bragg spot and can be used for structure solution only if methods are used that take dynamical diffraction into account.



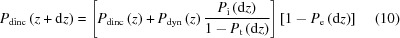
is the probability that dynamically scattered, incoherent electron scattered multiple times, always elastically, then it scattered one or more times inelastically. It will end up in a Bragg spot and can be used for structure determination if methods are used that take dynamical diffraction into account. However, because this electron experienced a thinner crystal coherently, dynamical corrections have to assume a thinner crystal, so the dynamical effect is less pronounced.



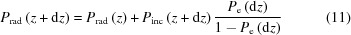
is the probability that electrons that first scattered inelastically and subsequently elastically. The normalization of *P*_e_(d*z*) by [1 − *P*_e_(d*z*)]^2^ takes account of multiple elastic events within the slice d*z*. The scattered wavefront originating from the location of the last inelastic scattering event exhibit a strongly pronounced scattering amplitude in the direction of the incident electron. Upon further elastic scattering events, this electron will not interfere with other elastically scattering electrons, hence the electron will not end up in a Bragg spot. Instead, it has a radially diffuse scattering probability. It will be considered as background by data integration programs.



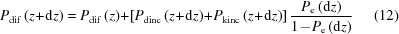
is the probability that an incoherent electron with a radially asymmetric diffuse scattering distribution first scattered elastically at least one time, then it scattered inelastically, rendering it incoherent. Subsequent elastic scattering diffuses its probability distribution according to a convolution of a radially symmetric distribution with the Bragg diffraction pattern. It will be considered as background by data integration programs.

**Figure 3:**
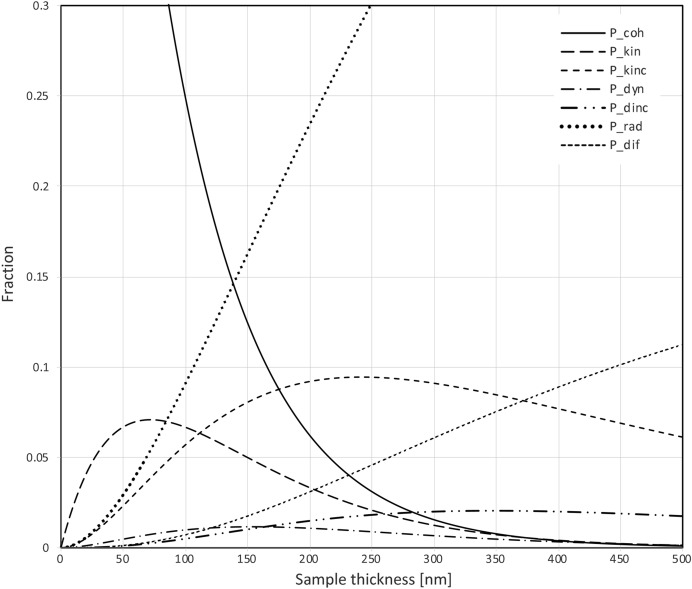
Probabilities of the various types (summarized in Fig. 2 ▸) of a 200 keV electron scattering in a hydrated protein sample. For other electron energies the horizontal axis has to be rescaled by a factor of 0.4835(*c*/ν)^2^. For 100 keV, 120 keV, 300 keV, 500 keV and 1000 keV, the rescaling factors are, therefore, 0.62, 0.71, 1.25, 1.42 and 1.83, respectively. So, for instance at 100 keV, the peak of *P*_kin_(*z*) occurs at a thickness of approximately 45 nm (0.62 × 70 nm), instead of at 70 nm.

The probabilities were calculated as a function of sample thickness *z*, using a finite difference method starting from *z* = 0, where *P*_coh_(0) = 1, and all the other probabilities are zero: *P*_inc_(0) = *P*_kin_(0) = *P*_dyn_(0) = *P*_inc_(0) = *P*_rad_(0) = *P*_dif_(0) = 0. Then, the thickness was increased to *z* + d*z* → d*z* and the probabilities were re-calculated. Next, the thickness was increased to *z* + d*z* → 2d*z* and the probabilities were re-calculated from the probabilities at d*z*, and so forth. The obtained distributions are shown in Fig. 3 ▸. Because the total scattering cross sections are linearly proportional to (*c*/ν)^2^, the shapes of these probability curves are constant, except for a scale factor (*c*/ν)^2^ for the axis corresponding to the sample thickness.

**Figure 4:**
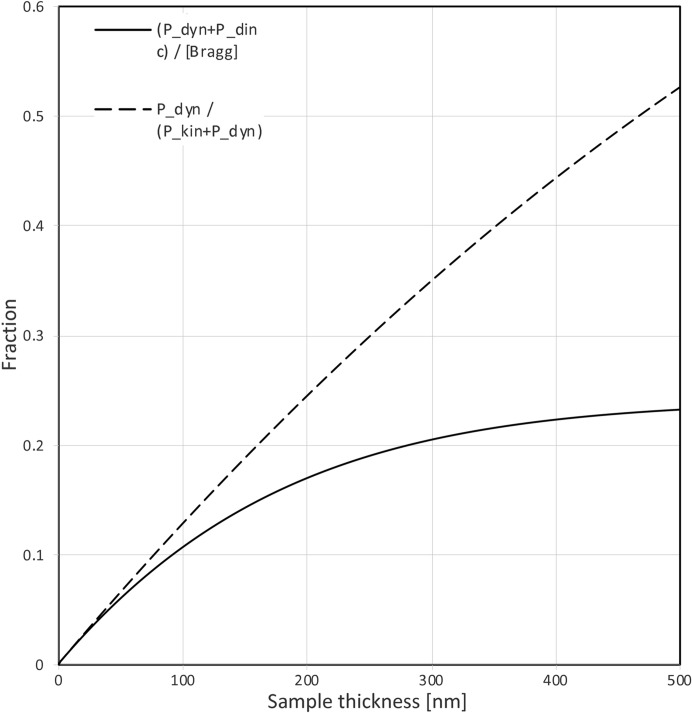
The solid curve corresponds to the calculated dynamical fraction of intensity in a Bragg spot as a function of sample thickness, taking inelastic scattering into account and assuming a 200 keV electron. The dashed curve assumes there is no inelastic scattering (or that the data have been measured using an energy filter and inelastically scattered electrons have been removed). The horizontal axis must be rescaled as explained in the legend to Fig. 2 ▸ for other electron energies. The asymptote of the solid curve is about 0.25, indicating that irrespective of the sample thickness, the kinematical fraction of a Bragg spot corresponds to at least 75% of the total intensity. The dashed curve eventually reaches unit, indicating that for very thick samples, the intensity in the Bragg spots becomes exclusively dynamical.

Here, we only consider the probabilities of events that end up in Bragg spots: *P*_inc_(*z*) + *P*_kinc_(*z*) account for the kinematic signal in a Bragg spot, whilst *P*_dyn_(*z*) + *P*_dinc_(*z*) account for the dynamical effect in a Bragg spot. We assume that the diffuse signals *P*_rad_(*z*) and *P*_dif_(*z*) can be removed by the data integration program. Even when the probability of diffuse scattering is (much) higher than the probability of Bragg scattering, it may not deteriorate the quality of the data very much, because the diffuse signal is spread over a much larger area than the Bragg signal. In Fig. 4 ▸, we consider the effect of ignoring inelastic scattering on the dynamical fraction of the Bragg signal, ignoring diffuse scattering.

We conclude from these calculations, that inelastic scattering reduces the fraction of dynamically scattered electrons in Bragg spots. It may therefore be beneficial to collect data of thick crystals without an energy filter. This may seem paradoxical, as neither the purely kinematical, nor the dynamical signal are reduced by an energy filter, as they resulted exclusively from elastic scattering events. However, an energy filter would remove electrons that first scattered elastically (one or more times) and that subsequently lost energy due to one or more inelastic events. Without an energy filter, these electrons would still end up in or very close to Bragg spots and could be used for structure determination and refinement. So filtering out these electrons would reduce the useful signal. Furthermore, our calculations indicate that the incoherent kinematical Bragg signal is much stronger than the incoherent dynamical Bragg signal [*P*_kinc_(*z*) ≫ *P*_dinc_(*z*); see Fig. 3 ▸]. Removing the incoherent Bragg scattering with an energy filter would therefore reduce the ratio between the integrated kinematical diffraction and the integrated dynamical from [*P*_kin_(*z*) + *P*_kinc_(*z*)]/[*P*_dyn_(*z*) + *P*_dinc_(*z*)] to *P*_kin_(*z*)/*P*_dyn_(*z*).

### 2.2. Effects of bulk solvent elastic scattering in multislice simulations   

In this section we treat electron scattering as a wave phenomenon. In multislice calculations, the sample is divided into slices normal to the direction of the electron beam. For each slice, the projected potential is calculated. Each slice diffracts the beam that passes through, according to its projected potential. So, the beam that hits a slice has been modified by all the previous slices. This series of Fresnel diffraction calculations cumulates into the ‘exit beam’ wavefunction when the beam leaves the final slice (Cowley & Moodie, 1957 ▸). We did not include the absorption potential of the sample in these multislice calculations, since this was also not considered by Glaeser & Downing (1993 ▸), and here we compare between including and excluding the effect of bulk solvent using their approach. The simulated scattering potential of protein and disordered water was only about 5 nm thick. Based on the average inelastic cross section of hydrated protein, a 5 nm thick sample would correspond to a loss of overall coherency due to inelastic effects of only about 7%. Ignoring the absorption potential of the sample therefore overestimates the ratio between kinematic and dynamical scattering, but only a fraction of 3 to 4%. We also did not include Ewald sphere curvature in our comparison between Friedel mates in our multislice calculations, as this was also not considered by Glaeser & Downing (1993 ▸). We are aware that excluding the Ewald sphere curvature in multislice calculations will inflate the apparent *R*_Friedel_ as a function of resolution. However, this effect is additive and is unaffected by the presence of absence of scattering potential of disordered water. The diffraction pattern is calculated by taking the Fourier transform of this exit wave and replacing its resulting complex values with their squared amplitudes.

In order to simulate the effect of including elastic scattering by the bulk solvent on the level of observable dynamical scattering, as monitored by differences within Friedel pairs, the following protocol was applied:

(1) Bacteriorhodopsin (trimer) atomic coordinates were downloaded from Protein Data Bank (PDB) entry 1brd (Henderson *et al.*, 1990 ▸).

(2) We assumed an orthorhombic unit cell of 63 × 63 × 52 Å^3^.

(3) The sequence of atoms was re-arranged in order of increasing *z*-coordinate, and numbered as a1, a2… .

(4) An incident plane wave with unit amplitude was assumed.

(5) The coordinates of the first atom a1 were read out from text file as 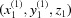. The chemical origin of the atom is *A*_1_.

(6) The transmission function in plane *z*_1_ was calculated as 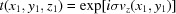, where σ is the interaction parameter and *v*_*z*_(*x*_1_, *y*_1_) is the projected 200 keV electron scattering potential of atom a1, calculated as described by Kirkland (2010 ▸) from the tabulated parameters corresponding to the chemical origin of *A*_1_. The transmission function and the complex-valued wavefront distributions were sampled with 630 × 630 pixels, with a pixel size of 0.1 Å. The exit wave in plane (*x*_1_, *y*_1_, *z*_1_) was calculated as

(7) *U*′(*x*_1_, *y*_1_, *z*_1_) = *t*(*x*_1_, *y*_1_, *z*_1_).

(8) The *z*-coordinate of the next atom a2 was read out: *z*_2_. The distance Δ*z* = *z*_2_ − *z*_1_ was calculated.

(9) The wavefunction *U*′(*x*_1_, *y*_1_, *z*_1_) was propagated for Δ*z* by employing the angular spectrum method (Kirkland, 2010 ▸). The resulting wavefront is *U*_2_(*x*_2_, *y*_2_, *z*_2_).

(10) The wavefunction was propagated atom by atom through the sample by repeating steps 6 to 8 until all atoms had been taken into account.

**Figure 5:**
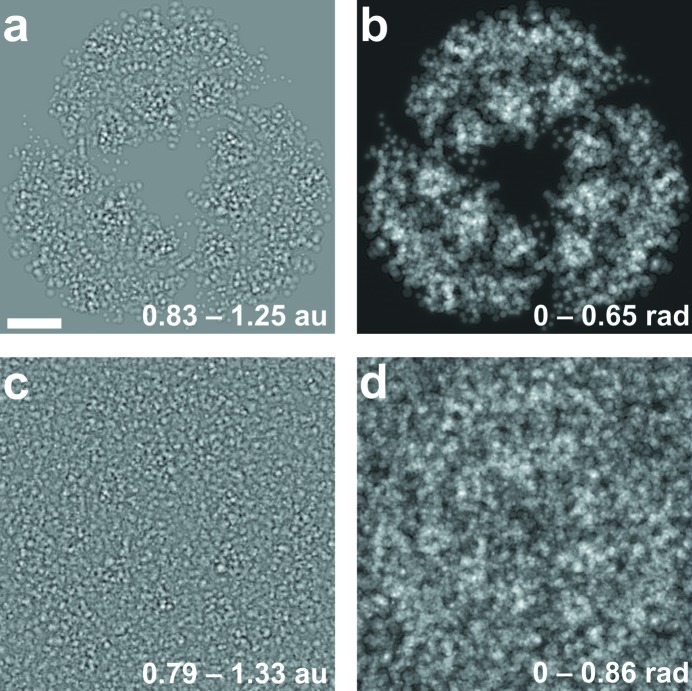
Distributions of electron exit wave which passed through the bacteriorhodopsin trimer without (top) or with (bottom) the inclusion or randomly placed water molecules, demonstrating the effect of bulk solvent scattering (the scale bar is 1 nm). The left panels show the amplitudes of the exit wave, the left panels show the phase of the exit wave. The transmission functions and the wavefunctions during the multislicing simulations were sampled with an accuracy of 0.1 Å.

In order to simulate the effect of bulk water, a random distribution of randomly rotated water molecules within the three-dimensional volume used for the multislice calculations was created based on an average density of water of about 33 molecules per nm^3^. Then the atoms of the bacteriorhodopsin trimer were inserted and the water molecules with coordinates that were located within 1.5 Å of any atom of the bacteriorhodopsin trimer were removed. The amount of the remaining water molecules corresponded to solvent content of about 70%. Next, the coordinates of all the atoms of the bacteriorhodopsin trimer and of the water molecules were listed all together as (*x*, *y*, *z*) coordinates in order of increasing *z*-coordinate, and numbered as a1, a2… . The mulstilsice simulations were done as described above in steps (4)–(10). The simulations were repeated 10 times with different random distribution of water molecules, and the resulting distributions were averaged. Fig. 5 ▸ shows the amplitude and phase distributions of the calculated exit wave, where in the simulation bulk solvent scattering was excluded (Fig. 5 ▸, top) or included (Fig. 5 ▸, bottom). The diffraction pattern was calculated as square of the amplitude of the Fourier transform of this exit wave (not shown).

**Figure 6:**
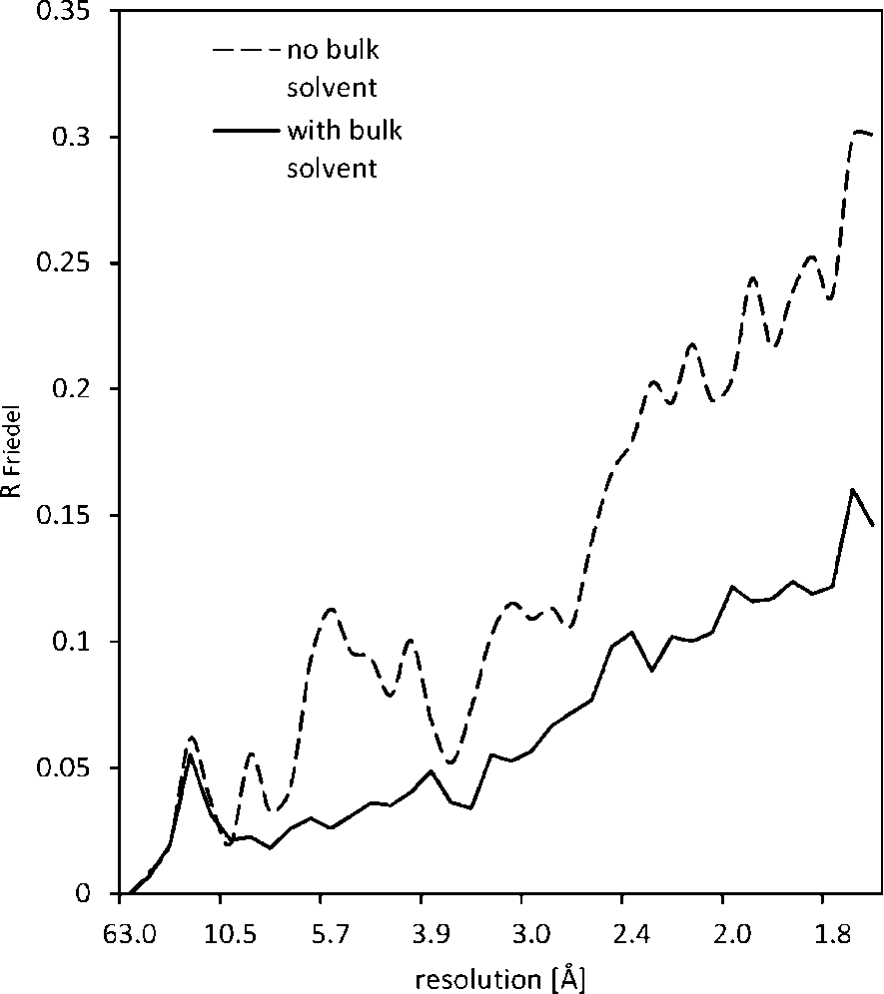
The effect of including bulk water in multislice calculations of 200 keV electron scattering within a protein sample on differences within Friedel pairs, as a function of resolution (in Å). The vertical axis is *R*_Friedel_(|*k*|), calculated over resolution bins as indicated in the text.

Glaeser & Downing (1993 ▸) calculated the differences within Friedel pairs due to dynamical electron scattering in a multislice simulation of the same bacteriorhodopsin atomic coordinates that we used for our simulations. However, unlike our simulations, these earlier calculations did not assess the contribution of disordered bulk solvent molecules on the magnitude of dynamical scattering. In order to quantify this effect, Glaeser and Downing used the non-standard *R*_Friedel_ statistics. In X-ray crystallography, differences within Friedel pairs are quantified by *R*_anom_ (Dauter, 2006 ▸): 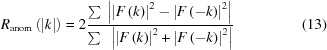
Though mathematically not equivalent, within the range of *R*_Friedel_ in Fig. 6 ▸, the difference between *R*_Friedel_ and *R*_anom_ is negligible. If Ewald sphere curvature is ignored, at low resolution they are equivalent, whilst at 2 Å resolution *R*_anom_ = 0.9*R*_Friedel_. The *R*_Friedel_ statistic is calculated from the two-dimensional Fourier transform of the exit wave and (unlike *R*_anom_) is not calculated from a three-dimensional data set. *R*_Friedel_ therefore ignores Ewald sphere curvature. It therefore inflates differences within Friedel pairs.
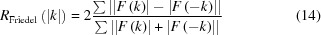
The *R*_Friedel_(|*k*|) statistic of our multislice calculations is plotted as a function of resolution in Fig. 6 ▸. The three-dimensional near-equivalent of *R*_Friedel_(|*k*|) that takes Ewald sphere curvature into account is *R*_anom_(|*k*|). In practice, the  *R*_anom_(|*k*|) of experimental protein and organic crystal diffraction data turns can be much lower than the *R*_Friedel_(|*k*|) based on multislice calculations (*e.g.* Clabbers *et al.*, 2019 ▸). This indicates that Ewald sphere curvature should not be ignored. Also, crystal bending and other types of disorder that are difficult to model in multislice calculations, and that will flatten out the electron scattering potentials, are prone to further reduce the ratio between the experimental  *R*_anom_(|*k*|) and theoretical *R*_Friedel_(|*k*|). Thus, these latter effects are likely contributors to reducing the apparent dynamical effect in experimental data (Subramanian *et al.*, 2015 ▸). According to our simulations, *R*_Friedel_(|*k*|) is less than 10% at low resolution and increases to about 40% at 2 Å resolution if bulk solvent scattering is excluded. However, *R*_Friedel_(|*k*|) increases only to half this value (20%) if bulk solvent is included. The average *R*_Friedel_ over this resolution range is 20% when bulk water scattering is excluded and 10% if bulk solvent is included in the simulations. Thus, excluding the scattering contribution of the bulk solvent, inflated the differences within Friedel pairs by a factor of two in our calculations.

## 3. Discussion and conclusions   

We have demonstrated that two effects that are usually excluded from multislice calculations of high-energy electron diffraction by protein samples can have a significant effect on the outcome of such simulations. The exclusion of the scattering potential of disordered bulk solvent results in a severe over-estimation of the dynamical scattering effects. For protein crystals with a thickness of several hundred nanometres, the effect of inelastic scattering also significantly contributes to a reduction in dynamical scattering, compared to naïve theory ignoring inelastic effects. Our results explain the discrepancy between theory and practice in protein crystallography. Here, the theory predicted electron diffraction of three-dimensional protein crystallography would not be possible because of dynamical scattering. However, in practice, it could be successfully realized even when crystals were up to 500 nm thick (*e.g.* Shi *et al.*, 2013 ▸). We assume our results may also impact on single particle cryo-EM, especially upon reducing the electron energy from 300 keV to 100 keV, which recently was shown to reduce inelastic scattering as a fraction of elastic diffraction (Peet *et al.*, 2019 ▸). At 100 keV electron energy, multiple elastic and inelastic events are about twice as likely, compared to 300 keV electrons, so multiple scattering events may also have to be taken into account for single particle structure determination.

## Figures and Tables

**Table 1:** Atomic elastic and inelastic scattering total cross sections in Å^2^ (σ_e_ and σ_i_, respectively) of an average atom of a hydrated protein or nucleic acid, as a function of the energy of the impinging electron

keV scattered e^−^	100	120	200	300	500	1000
σ_e_ protein	0.0042	0.0036	0.0026	0.0021	0.0017	0.0014
σ_e_ nucleic acid	0.0047	0.0041	0.0035	0.0029	0.0024	0.0021
σ_i_ protein	0.0174	0.0152	0.0108	0.0087	0.0070	0.0059
σ_i_ nucleic acid	0.0181	0.0158	0.0135	0.0113	0.0090	0.0079

## Footnotes

1 We define ‘electron scattering’ as any phenomenon that changes energy, phase and/or momentum of an electron, and reserve the term ‘electron diffraction’ exclusively for elastic events, which essentially preserve the energy of the electron.

## References

[bb1] Childs, P. A. & Misell, D. L. (1972). *J. Phys. D Appl. Phys.* **5**, 2095–2104.

[bb2] Clabbers, M. T. B. & Abrahams, J. P. (2018). *Crystallogr. Rev.* **24**, 176–204.

[bb3] Clabbers, M. T. B., Gruene, T., van Genderen, E. & Abrahams, J. P. (2019). *Acta Cryst.* A**75**, 82–93. 10.1107/S2053273318013918 PMC6302931 30575586

[bb4] Clabbers, M. T. B., van Genderen, E., Wan, W., Wiegers, E. L., Gruene, T. & Abrahams, J. P. (2017). *Acta Cryst.* D**73**, 738–748. 10.1107/S2059798317010348 PMC5586247 28876237

[bb5] Cowley, J. M. & Moodie, A. F. (1957). *Acta Cryst.* **10**, 609–619.

[bb6] Dauter, Z. (2006). *Acta Cryst.* D**62**, 867–876. 10.1107/S0907444906023481 16855302

[bb7] Egerton, R F. (2011). *Electron Energy-Loss Spectroscopy in the Electron Microscope*. Springer.

[bb22] Genderen, E. van, Li, Y., Nederlof, I. & Abrahams, J. P. (2016). *Acta Cryst.* D**72**, 34–39. 10.1107/S205979831502149X PMC4756612 26894532

[bb9] Glaeser, R. M. & Downing, K. H. (1993). *Ultramicroscopy*, **52**, 478–486. 10.1016/0304-3991(93)90064-5 8116103

[bb10] Hattne, J., Reyes, F. E., Nannenga, B. L., Shi, D., de la Cruz, M. J., Leslie, A. G. W. & Gonen, T. (2015). *Acta Cryst.* A**71**, 353–360. 10.1107/S2053273315010669 PMC4487423 26131894

[bb11] Henderson, R. (1995). *Q. Rev. Biophys.* **28**, 171–193. 10.1017/s003358350000305x 7568675

[bb12] Henderson, R., Baldwin, J. M., Ceska, T. A., Zemlin, F., Beckmann, E. & Downing, K. H. (1990). *J. Mol. Biol.* **213**, 899–929. 10.1016/S0022-2836(05)80271-2 2359127

[bb13] Howie, A. (1966). *Philos. Mag.* **14**, 223–237.

[bb14] Kantardjieff, K. A. & Rupp, B. (2003). *Protein Sci.* **12**, 1865–1871. 10.1110/ps.0350503 PMC2323984 12930986

[bb15] Kirkland, E. J. (2010). *Advanced Computing in Electron Microscopy*. Springer US.

[bb16] Loane, R. F., Xu, P. & Silcox, J. (1991). *Acta Cryst.* A**47**, 267–278.

[bb17] Nederlof, I., Li, Y. W., van Heel, M. & Abrahams, J. P. (2013). *Acta Cryst.* D**69**, 852–859. 10.1107/S0907444913002734 23633595

[bb18] Op de Beeck, M. & Van Dyck, D. (1996). *Ultramicroscopy*, **64**, 153–165.

[bb19] Peet, M. J., Henderson, R. & Russo, C. J. (2019). *Ultramicroscopy*, **203**, 125–131. 10.1016/j.ultramic.2019.02.007 PMC6495108 30773415

[bb20] Shi, D., Nannenga, B. L., Iadanza, M. G. & Gonen, T. (2013). *eLife*, **2**, e01345. 10.7554/eLife.01345 PMC3831942 24252878

[bb21] Subramanian, G., Basu, S., Liu, H. G., Zuo, J. M. & Spence, J. C. H. (2015). *Ultramicroscopy*, **148**, 87–93. 10.1016/j.ultramic.2014.08.013 25461585

